# Transplantation of Teeth

**Published:** 1887-04

**Authors:** Wm. N. Morrison

**Affiliations:** St. Louis, Mo.


ARTICLE IV.

### TRANSPLANTATION OF TEETH.

BY WM. N. MORRISON, D. D. S., ST. LOUIS, MO.

[Read in the Section on Dental and Oral Surgery at the Thirty-seventh Annual Meeting of the American Medical Association.]

I will give the history of one very interesting case. In the *Missouri Dental Journal*, of July 15, 1882, is a paper by me upon this subject, and among other cases reported is a prospective case which presented itself last fall: A young lady of slight build, about 22 years of age, teeth not of extra quality, arch perfect, and articulation correct, except the left-erratum; read right- instead of left-side of the mouth; superior canine, which was missing. The left-central was very loose. On careful examination I found it to be caused by the absorption of the root below the cervical line, by the crown of the canine, which lay horizontally in the process with its point to the median line, entirely across the root of the central, the latter being as thoroughly absorbed as though it was a deciduous tooth.

I had decided to extract both and transplant the canine to the socket of the central, after enlarging and deepening it to correspond to the root of the canine; after the canine had become fixed, if the crown could not be made to look as well as a central, I intended to cut it off and substitute a porcelain crown with a metallic pivot; but this heroic operation was indefinitely postponed by reason of the young lady being called home from our city.

This case is all the more wonderful from the fact that six dentists had already identified themselves with it; two of a neighboring city told her it had been broken off by a blow or a fall. Although previous to that decision one had constructed and adjusted regulating plates which were worn for months; exostosis being assigned as reason for enlargement; but a tooth never until I demonstrated it. Such was the case up to the time I first saw it, and gave my diagnosis and plan of operation. Other members of the patient's family had their favorite dentists, and she was taken to one and my plan duly explained and criticised; it was pronounced an impossible, impracticable and dangerous thing, but my diagnosis was pronounced correct so far as the canine was concerned, and its extraction was urged; to make this decision more forcible another was called in consultation, and they administered ether, and gouged away the process, exhausting themselves and the patient after breaking off a piece of the crown.

Further operations were postponed till some days later, when Dr. ———, who was called to use his persuasive powers and hold her hands, renewed hostilities, but with no better success. About three weeks later she was taken by the above or by their order to Dr. ———, who administered gas and extracted the canine, which was laid away to dry, and the patient assured that the central would grow firm again and be as useful as ever. When the root was entirely absorbed and the crown only suspended by slight attachment to the gum, the patient returned to her home, carrying with her the extracted canine trophy. All this occurred in the fall of 1881, and I had no knowledge of it until the discussion of my paper in the St. Louis Dental Society.

Now two years later, or three years after I first saw the case, the patient came to see if I would not transplant her right lower canine in the place of the upper central. The canine was condemned to extraction by another dentist, as its occlusion against the superior lateral was forcing the lateral out of place and making it assume an unsightly angle to the others. The central was hanging loosely to the gum and had been for three years, the other teeth supporting it laterally, and the lip and tongue antero-posteriorly; the patient was in constant dread of swallowing it, particularly at night. Contrary to the opinion of my brother dentists in the society, and statistical evidence collected from best dentists all over the country, (see discussion of the paper, *Missouri Dental Journal*, page 247, July 15th, 1882), the operation was performed; the loose tooth was removed and with a drill, (using cocaine). The process was opened up exactly in the track of the root which should have been there; at any rate I expected to find a piece of the root, but not a particle was there; with drill and fissure bur I enlarged and deepened the socket quite to the floor of the nose, and antero-posteriorly from plate to plate; and remember the anterior plate was cicatrical bone and not any to spare at that, owing to former extractions of canine, above described; the teeth and general shape of the socket being now known. The right lower canine was extracted, and root vessels removed, and canal filled with gold wire, and a prominent contour filling built upon its distal face or long angle, with platinum gold, which made the median edge of the new right central which it was to be. The root was long, and instead of being round as its predecessor, was oval or flattened on the sides, it was a fortunate thing that there was a thin scale of plate left upon the labial side when the socket was enough enlarged to receive the root.

A black rubber splint capping the crowns was worn about four weeks, shielding that tooth from injury; no unusual results followed; it was speedily taken into fellowship without an unfavorable symptom. The right lower first bicuspid was much within the arch, and by forcing it out with a jack-screw it performed excellent service as a canine. By this operation, although not as good as the one first contemplated, this beautiful young lady is spared the uncomfortable and humiliating necessity of wearing an artificial appliance, and has her own home-grown natural teeth distributed where they will do the most good.—*Journal of the American Medical Association.*

